# Flipper strokes can predict energy expenditure and locomotion costs in free-ranging northern and Antarctic fur seals

**DOI:** 10.1038/srep33912

**Published:** 2016-09-23

**Authors:** Tiphaine Jeanniard-du-Dot, Andrew W. Trites, John P. Y. Arnould, John R. Speakman, Christophe Guinet

**Affiliations:** 1 Department of Zoology and Marine Mammal Research Unit, Institute for the Oceans and Fisheries, 2202 Main Mall, AERL, University of British Columbia, Vancouver, BC, V6T1Z4, Canada; 2 Centre d’Etudes Biologiques de Chizé, CNRS, 79360 Villiers en Bois, France; 3 Deakin University, School of Life and Environmental Sciences (Burwood Campus), Geelong, Australia; 4 The Institute of Biological and Environmental Sciences, Zoology Bldg, Tillydrone Avenue, Aberdeen, AB24 2TZ, UK

## Abstract

Flipper strokes have been proposed as proxies to estimate the energy expended by marine vertebrates while foraging at sea, but this has never been validated on free-ranging otariids (fur seals and sea lions). Our goal was to investigate how well flipper strokes correlate with energy expenditure in 33 foraging northern and Antarctic fur seals equipped with accelerometers, GPS, and time-depth recorders. We concomitantly measured field metabolic rates with the doubly-labelled water method and derived activity-specific energy expenditures using fine-scale time-activity budgets for each seal. Flipper strokes were detected while diving or surface transiting using dynamic acceleration. Despite some inter-species differences in flipper stroke dynamics or frequencies, both species of fur seals spent 3.79 ± 0.39 J/kg per stroke and had a cost of transport of ~1.6–1.9 J/kg/m while diving. Also, flipper stroke counts were good predictors of energy spent while diving (R^2^ = 0.76) and to a lesser extent while transiting (R^2^ = 0.63). However, flipper stroke count was a poor predictor overall of total energy spent during a full foraging trip (R^2^ = 0.50). Amplitude of flipper strokes (i.e., acceleration amplitude × number of strokes) predicted total energy expenditure (R^2^ = 0.63) better than flipper stroke counts, but was not as accurate as other acceleration-based proxies, i.e. Overall Dynamic Body Acceleration.

Determining energetic costs of foraging is essential for understanding optimal foraging and the fitness of animals, yet is difficult to attain. Over the years, different techniques have been developed to determine energy expenditure, such as direct or indirect calorimetry techniques, breathing rates^1^, heart rates^2^, and locomotion speed^3^. While these techniques can be accurate in terrestrial or flying animals, they are usually either impossible to perform in the field or are overall poor predictors in diving species because of confounding factors inherent to diving physiology and biomechanics^4^, or changes in buoyancy and gliding^5^. As most of the metabolic cost of foraging comes from the cost of transport^6^ (i.e., the energy needed to move a unit of mass over a distance, usually expressed in J/m or in J/kg/m), count and amplitude of ‘stride’, ‘wingbeats’, and ‘stroke’ rates (in the case of marine animals) have been proposed as proxies of energy expenditure in a wide range of species^7^,^8^,^9^,^10^. Several methods such as video images^7^,^11^,^12^ or acoustic recordings^13^ can be used to record these stroke rates in aquatic animals, but they do not allow the intensity or amplitude of strokes to be estimated. More recently, acceleration that can characterize both frequency (i.e. rate) and relative amplitude of strokes as an index of swimming intensity has been used to investigate the cost of transport and has proven to be a cost-effective means to record data over long periods^14^,^15^,^16^.

Marine vertebrates can use either caudal or pectoral propulsion^17^. Most fish, cetaceans (whales and dolphins) and phocids (seals) undulate their bodies and use lateral or vertical oscillations of their rear appendages in a 2-phase propulsive stroke pattern^18^, while otariids (fur seals and sea lions), turtles or penguins use their fore-flippers for propulsion in a 4-phase stroke pattern with no resulting distortion of the body^19^. The latter, thus, generate a horizontal thrust and vertical lift, while animals that use their back flippers or fins for locomotion generate side-to-side thrust from hind-appendages acting as hydrofoil over the full stroke cycle and a forward propulsion^20^. As biomechanical efficiencies and performances of locomotor types depend on the shape of the propulsive surfaces and the kinematics of thrust generation^19^,^20^, stroking cost for animals with a caudal propulsion are likely not applicable to animals with pectoral propulsion and *vice versa*.

Stroking rate has been successfully related to energetic measures of foraging effort in a few marine vertebrates. For example, it has been shown that rear-flipper strokes have a more predictable impact on energy expenditure during dives than the dive duration itself in Weddell seals (*Leptonychotes weddelli*)^21^. Tail beats also correlate with acceleration, which in turn correlate with metabolic rates in salmon^22^ and sharks^16^,^23^. Energetic cost per stroke has been similarly studied in bottlenose dolphins^24^ and a few species of phocid seals^8^,^25^,^26^, but there are only a couple of studies in otariids on California sea lions (*Zalophus californianus*)^7^,^27^. The relative paucity of stroking data for marine mammals likely reflects the difficulty of collecting — and of energetically quantifying — such information for large diving animals.

Most studies relating foraging effort to stroking rates of marine mammals have been performed either in captive controlled settings^7^,^27^ or on single types of activities such as continuous diving in species that spend little time at the surface^8^,^21^. However, the cost of strokes as a proxy for foraging energy expenditure has not been quantified in free-ranging marine mammals engaged in their full complement of natural activities. Nor has any study given consideration to how the amplitude of the strokes affects swimming energetics given that power delivered with each stroke shapes energetics of locomotion^28^.

Northern fur seals (*Callorhinus ursinus*) and Antarctic fur seals (*Arctocephalus gazella)* are pelagic species of otariids (thus, fore-flipper swimmers) that — unlike most phocid seals — spend a significant portion of their foraging time at the surface where they transit, rest, and groom. The proportion of time that otariids perform different activities either at depth or at the water surface likely impacts their total foraging effort (through changes in drag, gliding, buoyancy, etc.) as shown in other free-ranging marine vertebrates^13^,^29^. Similarly, the type of locomotor mode they use for aquatic transport (i.e., swimming or porpoising) is also likely to impact their energetic costs of foraging^30^. However, it is not known whether flipper stroke metrics can accurately reflect energy expenditure in fore-flipper swimmers such as otariids under free-ranging conditions and how it might change with different types of swimming behaviour or activity.

The primary goal of our study was to determine whether flipper strokes of northern and Antarctic fur seals could be accurately detected from acceleration data for different types of activities and gaits during foraging trips in wild conditions. Second, we aimed to determine their energetic cost per stroke and their cost of transport, and to assess whether stroke rates or amplitude could accurately predict at-sea energy expenditure at the full foraging trip level or during specific types of activities.

## Results

Of the 40 fur seals instrumented with biologging tags and injected with doubly-labelled water, we removed 7 animals that had inaccurate metabolic rate measurements from further analyses. In addition, 3 daily diary tags failed to record any data and 4 stopped recording before the end of the foraging trip. Consequently, sample size for analyses performed over the total foraging trip were N = 13 for Antarctic fur seals and N = 13 for northern fur seals. However, the 4 partial acceleration datasets were included in analyses at the activity level as energetics and flipper strokes could be assessed over the same timeframe of existing data. This brought the sample size to 15 individuals per species. All values provided in the result section are mean ± SE.

### Foraging behaviours and flipper stroke metrics

Mass of the animals, flipper length and width, foraging parameters, and energy expenditure during total foraging trip or during diving and transiting are detailed in Table 1. Both species had similar trip duration, distance traveled and time spent diving *versus* transiting, but Antarctic fur seals performed on average more dives of shorter duration than northern fur seals. Both species also had similar at-sea metabolic rates and similar energetic costs for foraging and for transiting.

Overall, seals from both species spent more energy diving than transiting (Table 1). During dive time, fur seals beat their flippers 21.5 ± 2.3 times per dive (*p* = 0.759 for between-species comparison), which corresponded to a stroke frequency of 0.44 ± 0.06 Hz for Antarctic fur seals and 0.37 ± 0.03 Hz for northern fur seals (*p* = 0.004) while swimming at depth. Similarly, flipper stroke frequency when animals were transiting at the surface (surface speed > 1 m/s) was 0.53 ± 0.03 for Antarctic fur seals and 0.35 ± 0.02 for northern fur seals (*p* < 0.001). Flipper stroke frequencies were similar between surface transiting and diving within species (all *p* > 0.2). Animals with smaller body mass had significantly greater stroke frequencies (*p* < 0.01) no matter the species, but mass-corrected frequencies still showed that Antarctic fur seals tended to beat their flippers more frequently than northern fur seals during dive or transit times (both *p* < 0.001 for between-species comparisons).

Traveling at the surface (when animals can porpoise) required fewer flipper strokes than traveling the same distance while diving at depth. Antarctic fur seal beat their flippers 270 ± 14 times on average to travel 1 km at the surface, while northern fur seal only beat them 190 ± 19 times to travel the same distance (*p* < 0.001). However, both species required the same amount of flipper strokes to swim 1 km under water, i.e., 496 ± 30 (478 ± 35 for Antarctic fur seals and 514 ± 48 for northern fur seals, *p* = 0.418). There were also no significant differences between species when values were mass corrected.

The relative acceleration amplitude per flipper stroke (indicative of the relative swimming effort or intensity per stroke) while diving at depth was greater for Antarctic fur seals (0.75 ± 0.02 m/s^2^) than for northern fur seals (0.46 ± 0.01 m/s^2^; *p* < 0.001). Acceleration amplitude of strokes was also greater at depth than while transiting at the surface for Antarctic fur seals (0.48 ± 0.01 m/s^2^) compared to northern fur seals (0.43 ± 0.01 m/s^2^, *p* < 0.007). This translated into a cumulative swimming intensity per min of diving at depth (sum of acceleration amplitude per flipper stroke) of 22.6 ± 1.5 m/s^2^/min for Antarctic fur seals and 10.26 ± 1.0 m/s^2^/min for northern fur seals (*p* < 0.001) which was greater than the intensity of transiting at the surface (15.0 ± 1.1 m/s^2^/min for Antarctic fur seals vs. 8.7 ± 0.5 m/s^2^/min for northern fur seals, *p* < 0.001).

### Flipper strokes as index of activity-specific energy expenditure

**Figure 1:**
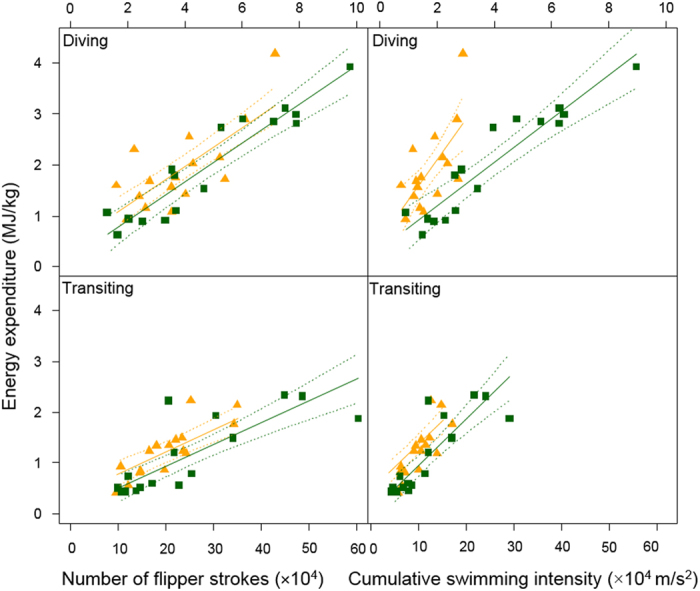
Relationships between number of flipper strokes during diving or transiting at the surface (2 left panels) or cumulative swimming intensity (from cumulative acceleration amplitude per stroke in m/s^2^, 2 right panels) and activity-specific energy expenditure (in MJ) for northern fur seals (orange triangles, N = 15) and Antarctic fur seals (green squares N = 15). Solid lines show the results of linear models (with species included as an independent variable), and dotted lines are the 95% CI on the predicted values.

The number of flipper strokes that fur seals beat while diving was a good indicator of energy spent to dive (R^2^ = 0.76, AIC = 46.4, *p* < 2 × 10^−10^, Fig. 1) according to the model:


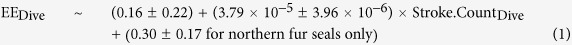


where EE is the energy spent while diving (in MJ/kg) and Stroke.Count_Dive_ is the total number of flipper strokes detected during diving. From this, we calculated that the energetic cost per flipper stroke at depth was 3.79 ± 0.39 J/kg/stroke for all seals (3.77 ± 0.47 J/kg/stroke for Antarctic fur seals and 3.84 ± 0.91 J/kg/stroke for northern fur seals, which did not differ significantly between the 2 species, *p* = 0.74). The relationship was also significant while transiting at the surface, but was slightly less accurate (R^2^ = 0.63, AIC = 36.4, slope *p* < 0.001, Fig. 1):


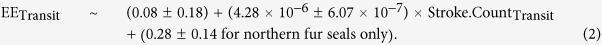


Finally, activity-specific cumulative stroke-acceleration amplitude correlated with activity-specific energy expenditure while diving and transiting (R^2^ = 0.66–0.72). Interestingly, the swimming acceleration amplitude (index of stroke intensity) while diving had a greater effect on energy expenditure for northern fur seals than for Antarctic fur seals 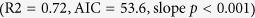, but this difference was not seen for surface transiting (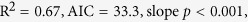 Fig. 1).


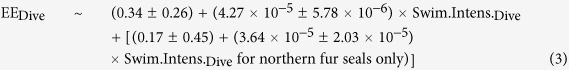



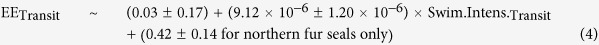


where EE_Dive_ and EE_Transit_ are energy spent in MJ/kg, and Swim.Intens._Dive_ and Swim.Intens._Transit_ are the cumulative swimming intensity in m/s^2^ during dive time and transiting time, respectively.

### Flipper strokes as index of energy expenditure over an entire foraging trip

**Figure 2:**
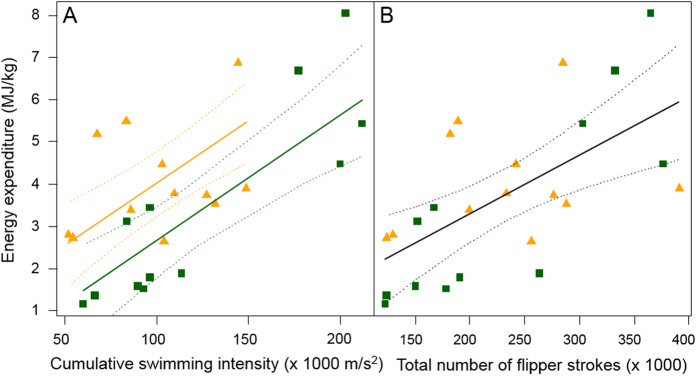
Relationships between cumulative swimming intensity (from cumulative acceleration amplitude per stroke in m/s^2^, graph (**A**) model 5 from Table 2, R^2^ = 0.63) or total number of flipper stroke during time at sea ((**B**) model 10 from Table 2, R^2^ = 0.50) over both diving and transiting periods and total energy expenditure (MJ/kg) for the foraging trip for female northern fur seals (N = 13, orange triangles) and Antarctic fur seals (N = 13, green squares). Solid lines are results of the general linear model and dotted lines are the 95% CI on the predicted values.

Over the entire foraging trip, the sum of all detected flipper strokes showed limited relationship with total energy expenditure at sea (R^2^ = 0.50, Fig. 2). The best models predicting total energy expenditures (Table 2 and Fig. 2) primarily involved swimming intensity (i.e., stroke acceleration amplitudes) while diving and transiting. The most parsimonious model (R^2^ = 0.63, AIC = 88.2) was:


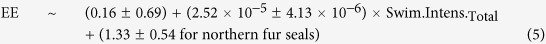


where EE is the total energy spent during foraging trip (in MJ/kg), Swim.Intens._Total_ is the cumulative swimming intensity while diving, and while transiting at the surface (sum of Swim.Intens._Dive_ and Swim.Intens._Transit_). Parameter estimates of other models are detailed in Table 2. It is interesting to note that only models involving swimming intensity (i.e., cumulative stroke acceleration amplitudes) showed a significant difference in the relationships between fur seal species, the ones involving flipper stroke count did not. Finally, rate of energy expenditure (in MJ/d) was not accurately predicted by flipper stroke counts or amplitudes (all R^2^ < 0.05).

### Cost of transport

Cost of transport (COT) calculated using the slope of the relationship between diving energy expenditure and vertical distance traveled was 1.63 ± 0.18 J/kg/m (*p* < 0.001, R^2^ = 0.71) in northern and Antarctic fur seals (not significantly different between species, *p* > 0.10). We also calculated a cost of transport of 1.88 ± 0.15 J/kg/m from the energetic cost per stroke (i.e., 3.79 J/kg/stroke) and the number of strokes necessary to travel one meter for each seal individually (no significant difference between species, *p* = 0.55).

## Discussion

Flipper strokes or tail beats are generally believed to be good proxies for the cost of transport and metabolic rates in fish and marine mammals^16^,^21^,^22^. In our study, we successfully determined the energetic cost of stroking (~3.8 J/kg/stroke) and the cost of transport in fur seals (1.6–1.9 J/kg/stroke). We also found that flipper stroke count was a good predictor of energy expenditure during diving (R^2^ = 0.76), and to a lesser extent during transiting (R^2^ = 0.63). However, flipper stroke count was a poor predictor overall of total energy spent during a full foraging trip at sea for fur seals (R^2^ = 0.50, Fig. 2). In this case, amplitude of flipper strokes (i.e., acceleration amplitude per stroke × number of strokes) represented total energy expenditure better than flipper stroke counts (best model in Table 2, R^2^ = 0.63), but was not as accurate as other acceleration-based proxies, such as Overall Dynamic Body Acceleration (ODBA)^31^ or Vectorial Dynamic Body Acceleration (VeDBA)^32^ whether per activity of over the full foraging trip^33^.

### Underwater locomotion

#### Energetic cost of stroking

**Figure 3:**
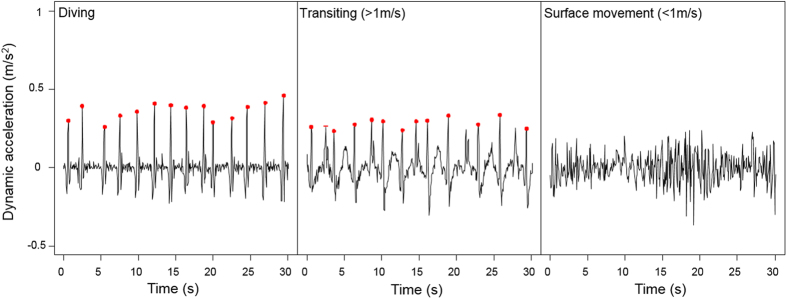
Examples of detection of fur seal flipper strokes from the dynamic acceleration on the X and Y accelerometer axes while diving, while transiting at the surface at speeds > 1 m/s, or while performing slower movements at the surface at speeds < 1 m/s.

Detecting flipper stroke count and relative amplitude was straightforward when animals were diving (Fig. 3) and allowed us to accurately predict energy spent while diving in both species of fur seals (Fig. 1), as was previously done in Weddell seals (R^2^ = 0.87^21^). From this, we calculated a cost per stroke for fur seals is ~3.8 J/kg/stroke while diving (with no significant difference between the two species). This value is similar to the 3–4 J/kg/stroke values found for northern elephant seals^8^ and falls within the range of values for other phocid seals (2.88 J/kg/stroke in harp seals to 5.74 J/kg/stroke for harbour seals^20^,^34^). Phocid seals mostly swim using their hind-flippers for pseudo-axial locomotion, while otariids use their fore-flippers for ‘underwater flight’ locomotion^35^. The difference in kinematics and generation of thrust between these two swimming modes results in different drag, stroke power and efficiencies, which could explain the observed ~ 2.5-fold difference in energy cost per stroke between taxa.

Buoyancy can also affect stroking patterns and the numbers of strokes required to cover a given distance in diving elephant seals^14^. This in turn affects locomotion costs in different ways depending on seal density and whether it descends or ascends. Locomotion costs are usually minimal with neutral buoyancy^36^, which can be attained at different pressures or depths depending on body fat, body size, quantity of internal gas, or to a lesser extent seawater temperature or salinity^37^. The northern and Antarctic fur seals in our study were positively buoyant and used their flippers at faster frequencies to maintain a constant swim speed during descent than ascent (visually assessed but not reported and consistent with previous observations^13^). Otariids have a lower ratio of fat to lean mass than phocid seals^38^. They also dive with fully inflated lungs and with air trapped in their fur, unlike phocid seals that exhale before diving and are devoid of thick fur^39^. Furthermore, otariids are on average shallower divers than phocid seals^39^, and might not dive deep enough to attain neutral buoyancy as elephant seals do^40^. This inherent difference in buoyancy while diving could also explain, at least partially, the difference in cost of stroking at depth between taxa.

The cost per stroke made by our fur seals was lower than the average 7.33 J/kg/stroke found in captive California sea lions, the only other study on otariids. However, our value fell within their large reported range of values (1.93–23.16 J/kg/stroke)^7^. These observed differences might be explained by variations in buoyancy, dive angle, dive depth, and capacity for gliding that all affect the cost of transport in marine predators^40^,^41^,^42^,^43^,^44^. In our study, northern and Antarctic fur seals were free to swim at chosen depths and did glide during dive phases. In contrast, the captive California sea lions swam in flumes and, thus, had no room for any behavioural adjustments. The difference between flume swimming in captivity and free-ranging swimming in open oceans could explain the ~1.8-fold difference on stroking cost between captive California sea lions and our 2 species of free-ranging fur seals.

#### Cost of transport

We calculated the underwater COT to be ~1.6–1.9 J/kg/m in northern and Antarctic fur seals based on the slope of the relationship between diving energy expenditure and vertical distance traveled, as well as the energetic cost per stroke and the number of strokes necessary to travel one meter. Our values are lower than COT for other ‘aquaflying’ animals such as Steller sea lions^45^, California sea lions^25^,^27^, penguins^46^,^47^, turtles^48^,^49^, and phocid seals, such as harbour or grey seals^25^,^26^,^34^. It was, however, slightly higher than the COT of ~1.2–1.4 J/kg/m for elephant seals calculated given a cost of ~3–4 J/kg per stroke^8^ and the 0.4 stroke necessary to cover 1 m (0.35–0.45 strokes^14^). The cost of transport (COT) in J/kg/m has been hypothesized to be consistent between marine mammals and to scale with body mass according to the equation^27^ COT = 7.79 × mass^−0.29^ . Our fur seal COT values are ~30% lower than the theoretical value from the above equation^27^ of 2.7 J/kg/m for a female fur seal of average mass. Similarly, elephant seals also have a 15–22% lower COT (~1.2–1.4 J/kg/m) than the 1.55 J/kg/m theoretical value for a 250 kg female elephant seal^27^.

Interestingly, the only otariid (California sea lion) included in the dataset used to derive the theoretical COT for all marine mammals^27^ also had a lower measured COT values compared to the predicted value (open circles in Fig. 1 in ref. 27). The two extreme ranges of body mass estimates between pinnipeds (15–100 kg) and cetaceans (2000–10000 kg) might have driven the regression equation between these 2 clusters, rather than resulting from an actual functional relationship between taxa. Pinniped values alone do not seem to follow a clear trend (Fig. 1 in ref. 27), so the derived relationship might not be accurate for this group. On the other hand, each pinniped study reported in ref. 27 was performed in swimming flumes, i.e., measured COT for a surface swimming with no possibilities of adjusting swimming speed and depth or of gliding which are all energy-saving mechanisms^38^,^40^. The theoretical COT equation could thus overestimate COT for free-ranging animals with more behavioural flexibility. This is confirmed by there being no significant difference between measured and theoretical values of COT in Steller sea lions (*Eumetopias jubatus*) swimming in a flume tank^45^, and that the only other COT estimate from wild marine mammal (i.e., for the elephant seals mentioned above) is also overestimated. Another possibility is that inherent errors in the detection of flipper strokes at sea from acceleration (although they are minimized during diving) and in calculated flipper stroke rate per distance might influence the accuracy of our measured COT in fur seals.

#### Differences between fur seal species

Energetics and efficiency of swimming depends on morphology of animals, on their total body surface area and on the propulsive power of their flippers^19^,^50^. Northern fur seals were slightly heavier and longer than Antarctic fur seals, but mostly had much longer and wider fore- and hind-flippers (Table 1). Surface area of the fore-flippers used for propulsion (calculated as twice the surface area of an ellipse to account for both sides of the flipper) was ~25% greater for northern fur seals than Antarctic fur seals, and hind-flipper surface area (calculated as twice the area of a triangle) ~50% greater. They, thus, constituted a greater proportion of total surface area than they do as a proportion of body mass or body length (i.e., ~18% and ~11% difference in body mass and length respectively).

As a consequence of having bigger flippers, northern fur seals could theoretically produce proportionately greater lift and thrust power while swimming than could Antarctic fur seals. However, the flippers of the northern fur seals also likely created more drag and required greater power^20^ given that drag increases with total body surface area of an individual^51^. These morphological differences might explain why northern fur seals beat their flippers less frequently on average than the Antarctic fur seals, and why they required a greater change in energy expenditure for a given change in flipper stroke intensity (estimated from acceleration amplitude in m/s^2^) than did the Antarctic fur seals (Fig. 1).

We had expected flipper stroke intensity generated while swimming underwater (an index of stroke power) to be greater for northern fur seals than for Antarctic fur seals given the differences in flipper morphology. However, we found the opposite to be the case (i.e., 0.75 ± 0.02 m/s^2^ for Antarctic fur seals vs. 0.46 ± 0.01 m/s^2^ for northern fur seals, Table 1). One possible explanation for this finding is that the measured acceleration is intrinsically greater per joule spent for Antarctic fur seals swimming at depth than for northern fur seals (different J/m/s^2^). This would be consistent with the energetic cost per stroke being similar between species, while the acceleration amplitude is greater per stroke for Antarctic fur seals which means that a change in acceleration should impact energy expenditure in Antarctic fur seals less rapidly than for northern fur seals (Fig. 1). However, this could result from the fact that diving metabolic rates were not found to differ between species.

Another possible explanation for Antarctic fur seals having higher flipper stroke intensities than northern fur seals is that the underwater behaviours of Antarctic fur seals required a different analytic method to filter the dynamic acceleration output during a flipper stroke in m/s^2^ compared to northern fur seals. Flipper stroke can vary depending on speed, drag (thus body morphology and swimming speed), and chasing behaviour. If different prey species targeted between species require different chasing speeds, this might impact the drag force that animals would have to counteract while stroking. In any case, differences in body morphology and kinematics of swimming did not affect the relationships between flipper stroke counts and energy expenditure while swimming, only the relationships between flipper stroke intensity and energetics during underwater swimming.

### Surface locomotion and full foraging trip energetics

Most studies that have linked energy expenditure to cost of locomotion through flipper stroke rates in the wild have done so for species that spend most of their time diving^8^,^21^. However, fur seals spend ~70% of their time at-sea at the surface, transiting, resting, and performing surface activities (grooming, slow movements). As energetic demands of swimming differ at the surface and depth due to physical properties of oceans and of animal behaviour^40^, the time-activity budget will impact energy spent over the full foraging trip.

Quantifying flipper stroke rates for fur seals at the surface was not as straightforward as at depth (Fig. 3) due largely to the environmental acceleration ‘noise’ from waves that could mask the dynamic acceleration signal of flipper stroke, and to the porpoising behaviour of seals. Unfortunately, the residual waves-related acceleration and change of mode of locomotion prevented us from estimating a biologically accurate energetic cost per stroke while transiting. However, it is clear from the slopes in Fig. 1 that it costs fur seals less to transit than it does to dive. This might be due to the fact that fur seals tend to transit at 3-body diameter depths where drag is the lowest^52^ or to porpoise to spare energy^40^. This is similar to what has been found in Antarctic fur seals^53^, but opposite to other results based on diving behaviour only (although fur seal transit time included resting and surface activity)^54^.

The best models to predict the total energy expended by fur seals during their foraging trips all included ‘swimming intensity’ during diving and transiting (i.e., relative amplitude of acceleration during stoking), rather than flipper stroke count. Number of flipper strokes better reflected energy expenditure at the activity level while acceleration amplitude of flipper strokes performed better at the foraging trip level. Since the relationships between energetics and flipper strokes differ between diving and surface transiting, the proportion of time individuals allocate to these activities will result in different amounts of energy being spent over the entire foraging trip. It is, therefore, logical that the acceleration amplitude of flipper strokes or swimming intensity correlates better with energy expended over the entire foraging trip than with stroke counts. Acceleration amplitude incorporates an index of difference in intensity between diving and transiting costs of transport that simple stroke counts cannot. Furthermore, diving and transiting only accounted for ~60% of total time at sea in fur seals. This means that animals spent ~40% of their time performing other activities (surface activities, slow movements, grooming, sleeping) overall less expensive than diving or transiting^55^. Whether animals do not stroke while performing surface activities, or whether we could not detect it amongst environmental movements is unclear. In any case, acceleration amplitude per flipper stroke seem to better account for these other surface activities than flipper stroke count, even though its overall accuracy as a predictor for total energy expenditure at sea remains limited (R^2^ = 0.63).

Insley^13^ suggested that multiplying the energetic cost per stroke at depth by an average stroking frequency was a good approximation for total energy spent at sea given that fur seals spend 70% of their time swimming (whether at depth or at the surface). However, this assumes that costs of transport at depth and at the surface are similar, which is not the case (Fig. 1). Northern and Antarctic fur seals in our study spent 20–46% of their time diving and 15–47% of their time transiting. These high intra-individual differences in time-activity budgets are important to consider when trying to estimate the cost of foraging given how different activities have different associated costs^33^.

Swimming intensity derived from the relative amplitude of flipper strokes is one way to measure dynamic body acceleration associated with locomotion and estimate energy expenditure. ODBA or VeDBA are other acceleration-based metrics that have been used to estimate energy expended by different species while foraging^16^,^56^,^57^,^58^,^59^,^60^. Overall, ODBA or VeDBA were better proxies for energy expenditure than flipper stroke metrics during specific types of activities performed (R^2^ > 0.85^33^). Flipper strokes only reflect one specific type of movement, while active swimming and foraging usually involves many other body motions (undulations, barrel-rolls, etc.). Furthermore, flipper strokes are only detected over 2 body axes (up/down and forward/backward) and are not reflected on the third axis, while ODBA/VeDBA are a measure of tri-axial acceleration. Finally, using ODBA/VeDBA took into account the types of surface movements that flipper strokes could not (~40% of the activity budget). It is, thus, not surprising that ODBA or VeDBA correlated better with energy expenditure either at the activity specific level or at the full foraging trip timescale, as it considers more body movements than just flipper strokes.

### Conclusion

Flipper stroke rate is an accurate means of determining the energy spent by fur seals while swimming underwater (i.e., while diving) as has been shown for other seals^7^,^8^,^21^. However, flipper stroke count or amplitudes are not the best predictors of total energy expenditure over a full foraging trip because of the time fur seals spend at the surface^55^ (due to porpoising behaviours and inherent difficulties in detecting flipper strokes at the surface). While morphology and gaits have undoubtedly evolved to maximise locomotion efficiencies in the ocean, there are sufficient behavioural differences between species and taxa to confound application of a simple metric of movement that can be universally applied. Rather, foraging ecology studies need to consider the activity-specific metabolic rates and time-activity behavioural strategies that ultimately determine the foraging costs of individuals^55^.

## Material and Methods

### Data collection

Data were collected from 20 lactating northern fur seal females at the Reef rookery on St Paul island (Bering Sea, 57°6′N–170°17′W) during the breeding season in Aug-Sep 2011, and from 20 lactating Antarctic fur seal females at Pointe Suzanne, Kerguelen Island (Southern Ocean, 49°26′S–70°26′E) during the breeding season in Jan–Feb 2012. Data were collected under the US NMFS permit #14329-01, the UBC animal care permit #A10-0364 and the ethical regulations approval from the French Polar Institute (IPEV). All methods were performed in accordance with the relevant guidelines and regulations stated by the permitting agencies.

All study females were healthy-looking adults and were confirmed to be provisioning a pup. They were captured using a hoop net, carried over a short distance to a restraint board where they were anaesthetized with isoflurane gas. Standard morphometric measurements of length, axial girth and flipper lengths and widths were made to the nearest 0.5 cm, and mass was recorded using scales with accuracy within ±0.2 kg. The seals were equipped with data loggers glued directly on their fur using a 2-part Devcon 5-min epoxy glue. Daily diary tags (Wildlife Computers) recording tri-axial acceleration at 16 Hz, depth, light level, and water temperature at 1 Hz were glued between the shoulder blades. Fastloc GPS MK10 loggers (Wildlife Computers) were glued lower down the back from the daily diary tags. They recorded GPS coordinates along the track of the animal at sea, as well as depth and water temperature at 1 Hz. Once the devices were securely attached, the females were released upon full recovery from the anaesthesia and allowed to rejoin the colony. The females were recaptured after a single foraging trip at sea and were anesthetised a second time using the same method as mentioned above. Data loggers were removed by cutting the fur, and blood samples and morphometric measurements were taken a second time.

### Diving/foraging parameters and time-activity budgets

Time activity budget of individual seals at sea was partitioned between diving, transiting (i.e., traveling at the surface at a speed ≥ 1 m/s), surface activity (time at surface at a speed < 1 m/s) and resting using methods detailed in ref. 55. In brief, we used depth data recorded by the DD tags to determine diving behaviours unless the tags malfunctioned and we thus used depth data recorded by the Fastloc MK10. Diving behaviours were reconstructed using a custom-made R program previously developed for Antarctic fur seals. Dives were defined as periods of time that animals spent under water below a minimum depth of 3 m and for a minimum of 4 s until they went back to the surface. It also included the post-dive intervals estimated using the Bout-Ending Criterion (BEC) from the maximum likelihood estimation method using the package diveMove in R (Author, S. Luque). Transiting and surface activities were defined as time when animals were not diving and traveled at speeds either above or below 1 m/s. Travel speed at the surface was calculated from the time it took to travel a given linear distance between two consecutive GPS locations taking into account the curvature of the Earth using the Haversine formula^61^. Resting time was calculated by applying a running variance over 3 s on the raw acceleration of each of the 3 axes and then defined as the time when the resulting acceleration variance signal was less than 2.5 m/s^2^ for all 3 axes for more than 5 min. This acceleration threshold was determined visually and was similar for all animals. Vertical distance traveled while diving was calculated by doubling the maximum dive depth of each dive, which should provide an acceptable estimate as fur seals are known to dive at relatively shallow depths (~35 m average).

### Energy expenditure at sea

Measurements of daily energy expenditure (DEE, kJ/day) were performed using the doubly-labelled water (DLW) method^62^,^63^. Details on the procedure can be found in ref. 55. Briefly, 3 blood samples were collected on the hind-flipper of the seal. The first blood sample was drawn upon capture to measure background level of O and H stable isotopes in the seals’ body. The 2^d^ blood sample was collected 2 h after the intravenously administered dose of DLW (0.3–0.6 g/kg body mass of 622272 ppm ^18^O, 384645 ppm ^2^H) was allowed to equilibrate in the body. The third blood sample was collected after the seal’s foraging trip at sea upon recapture. The isotope ratios ^18^O: ^16^O and ^2^H: ^1^H were analysed using gas source isotope ratio mass spectrometry (Optima, Micromass IRMS and Isochrom μG, Manchester, UK). Isotope enrichments were converted to CO_2_ production for each individual using a two-pool model, and initial isotope dilution spaces were calculated using the plateau method^64^. We used the equation from Speakman *et al*.^65^ to account for evaporation loss, and CO_2_ production rates were converted into daily energy expenditure using a respiratory quotient (RQ) of 0.80^66^,^67^.

Female fur seals spent some time on land after the post-equilibration sample and before we recaptured them for the third blood sample after their foraging trip. Energy spent during this ‘non-foraging’ time was part of the DLW measurement. However, we were interested in estimating only energy expenditure at sea. Metabolic rate of lactating females while on land has been measured in northern fur seals (4.67 W/kg in ref. 68) and Antarctic fur seals (4.56 W/kg in ref. 69). We, thus, subtracted the amount of energy that the females spent on land, based on the total time they were on land, from the total energy expenditure measured by DLW to obtain the energy spent at sea. The energy each animal spent diving or transiting were determined using their respective time engaged in these activities during their foraging trip multiplied by the activity-specific metabolic rates calculated in ref. 55 (diving metabolic rate of 30.84 MJ/d and transiting metabolic rate of 18.5 MJ/d for northern and Antarctic fur seals).

### Flipper strokes

Flipper strokes were detected and counted using only the X (surge) and Z (heave) accelerometer axes. Fur seals swim by flapping their fore-flippers which results in an up and forward movement clearly and synchronously visible on the 2 signals. The dynamic accelerations *X*_*dyn*_ and *Z*_*dyn*_ were calculated by using a running mean of 2 s^66^,^70^,^71^ to dissociate the static acceleration (due to the positioning of the animal in space in respect to gravity) from the dynamic acceleration (due to the movement of the animal). We then added *X*_*dyn*_ and *Z*_*dyn*_ to increase the signal clarity compared to residual background noise. Each flipper stroke was detected by counting the spikes resulting from a deviation from the gravitational field above a specific threshold (Fig. 3). Thresholds were determined for each animal individually as the breaking point of a curve showing the number of flipper stroke detected for a range of thresholds going from 0.1 to 0.4 m/s^2^. Thresholds ranged from 0.19 to 0.24 m/s^2^ depending on individuals. We assumed that fur seals have an absolute maximum flipper stroke frequency of 2.5 Hz, well above average flipper stroke rate reported for free-ranging otariids (0.4–0.6 Hz)^13^ and same as the maximum stroke rate found in a phocid species of similar mass^60^,^61^. We are aware that phocids have different propulsion methods compared to otariids but maximum stroke rate value could not be found for an otariid species, and we consider this to be a conservative value. Consequently, any peak within 0.4 s of a previous one was not categorized as a flipper stroke. As the dynamic acceleration is centered on 0, we also used the value at the highest point of the flipper stroke as a relative index of flipper stroke amplitude (indicative of swimming intensity).

**Figure 4:**
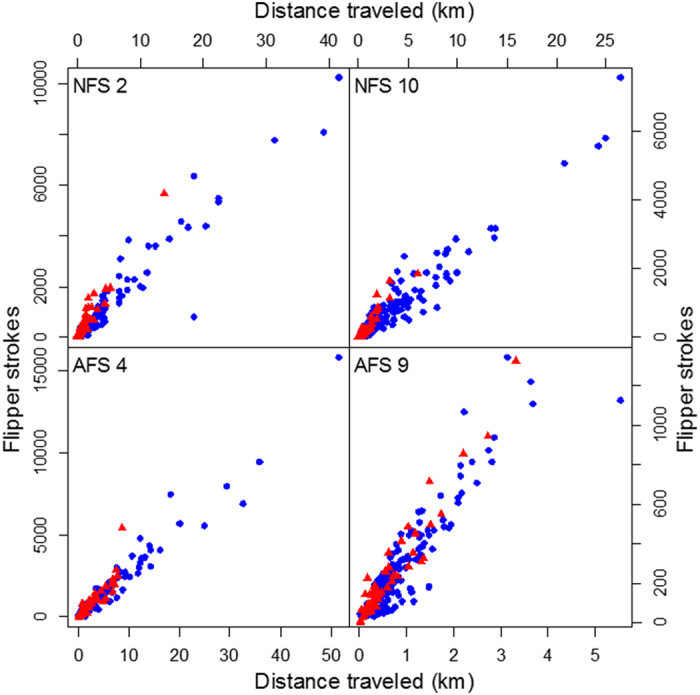
Number of flipper strokes detected per distance traveled vertically while diving (red triangles) and horizontally while transiting near the surface (blue circles) for 2 northern fur seals (NFS 2 and NFS10) and 2 Antarctic fur seals (AFS 4 and AFS 9) between 2 GPS locations during a foraging trip. Transiting at the surface is defined as periods of time when animals travel at speeds > 1 m/s between 2 GPS locations.

Flipper strokes where easily detected when animals were diving (Fig. 3). However, oceanic/wave movements often clouded the acceleration due to the movement of the animal when at the surface. We were only able to detect flipper strokes when animals were at the surface travelling at a minimum of 1 m/sec. Below this travel speed, the animal seemed to be either not stroking, or stroking with such a low amplitude that it was impossible to separate dynamic acceleration signal coming from oceanic movements or animal movements (Fig. 3). Consequently, we only measured flipper strokes at depth or at the surface when the animal was moving at or faster than 1 m/sec between 2 GPS points. Accuracy of peak threshold and flipper stroke detection were verified visually on 10 5-min periods randomly chosen over the entire foraging trip for each seal. Relationship between flipper strokes and distance traveled was also verified for each animal at depth and while transiting at a speed >1 m/s at the surface (examples in Fig. 4) to validate the detection method.

### Statistics

The number of flipper strokes or cumulative acceleration amplitudes of strokes (indicative of swimming intensity) during dives or between 2 GPS locations were statistically analyzed using linear mixed effects models with animal ID nested in species. Fixed effects were horizontal distances for number of flipper strokes while transiting at the surface and vertical distance for flipper strokes while diving. Autocorrelation in the data was corrected using the autoregressive moving average structure (lag 1) and deviance of heterogeneity of variances if any by a power structure.

Relationships between energy spent during total trip (or while diving and transiting) and number of flipper strokes or cumulative stroke acceleration amplitude were estimated using general linear models (lm, ‘stats’ package, R 3.0.3) or general linear model using generalized least squares that allows for unequal variances (gls, ‘nlme’ package, R 3.0.3) upon verification of model assumptions. These methods minimize the squared residuals in the dependent variable.

Cost of transport (COT) was estimated first from the slope of the relationship between diving energy expenditure and vertical distance traveled using a using general linear models (lm, ‘stats’ package, R 3.0.3) as mentioned above (in J/kg/m). Second, we also calculated cost of transport by multiplying the energetic cost per stroke (i.e., 3.79 J/kg/stroke) by the number of strokes necessary to travel one meter (stroke/m) for each individual.

Finally, we tested which associations of parameters (cumulative or average stroke amplitude, number of flipper strokes while diving, while transiting or total) would best predict energy expenditure at sea over a full foraging trip time-scale. We tested all variable combinations and selected best models based on the AICc (second-order information criterion or AIC adjusted for small sample size compared to the number of estimated parameters). We also computed AICc weights to provide information on the model probability given the data and a set of models where one of the models is considered the best model^72^.

## Additional Information

**How to cite this article**: Jeanniard-du-Dot, T. *et al*. Flipper strokes can predict energy expenditure and locomotion costs in free-ranging northern and Antarctic fur seals. *Sci. Rep.* **6**, 33912; doi: 10.1038/srep33912 (2016).

## Figures and Tables

**Table 1:** Morphometric, metabolic and foraging parameters for 13 female northern and 13 female Antarctic fur seals during a single foraging trip at sea.

Parameters	Northern fur seals		Antarctic fur seals	
Mass (kg)	37.9 ± 1.3*		31.1 ± 0.8*	
Length (cm)	128.1 ± 1.6*		114.5 ± 1.3*	
Axillary girth (cm)	78.7 ± 1.2		81 ± 1.2	
Fore-flipper length (cm)	39.4 ± 0.9*		34.3 ± 0.3*	
Fore-flipper width (cm)	12.5 ± 0.2*		11.1 ± 0.1*	
Hind-flipper length (cm)	34.6 ± 0.6*		24.4 ± 0.4*	
Hind-flipper width (cm)	14.4 ± 0.2*		10.8 ± 0.2*	
Trip duration (d)	7.96 ± 2.17		7.65 ± 3.88	
Distance traveled (km)	750 ± 50		635 ± 77	
Number of dives	2551 ± 323*		3949 ± 597*	
Dive duration (s)	62.8 ± 7.3*		42.6 ± 4.5*	
Time spend diving (%)	28.6 ± 2.0		29.0 ± 0.7	
Time spent transiting (%)	30.5 ± 1.8		26.4 ± 1.6	
Time spend diving (h)	53.9 ± 4.7		47.7 ± 6.0	
Time spent transiting (h)	59.9 ± 5.9		45.9 ± 7.7	
At-sea metabolic rate (MJ/d/kg)	0.56 ± 0.04		0.59 ± 0.04	
Total energy expenditure (MJ/kg)	4.2 ± 0.4		4.1 ± 0.6	
	**Northern fur seals**		**Antarctic fur seals**	
	**Diving**	**Transit**	**Diving**	**Transit**
Energy expenditure (MJ/kg)	1.89 ± 0.81	1.24 ± 0.51	2.00 ± 1.03	1.15 ± 0.75
Flipper stroke Frequency (Hz)	0.37 ± 0.03*	0.35 ± 0.02*	0.44 ± 0.06*	0.53 ± 0.03*
Swim. intensity/stroke (m/s^2^)	0.46 ± 0.01*	0.43 ± 0.01*	0.75 ± 0.02*	0.48 ± 0.01*
Swim. intensity/min (m/s^2^/min)	10.26 ± 1.0*	8.70 ± 0.52*	22.60 ± 1.51*	15.01 ± 1.13*

Values are mean ± SE and ^*^ show between species significant differences (*p* < 0.05). Swimming intensity refers to acceleration amplitude per stroke (m/s^2^) or per min of stroking (m/s^2^/min).

**Table 2:** Best models explaining at-sea energy expenditure in MJ/kg of northern (N = 13) and/or Antarctic fur seals (N = 13) as a function of flipper stroke count or of swimming intensity, i.e., cumulative acceleration amplitude of strokes during diving and transiting combined (Stroke.Count_Total_ and Swim.Intens._Total_), or while diving or transiting at the surface (Stroke.Count_Dive,_ Stroke.Count_Transit,_ Swim.Intens._Dive_ and Swim.Intens._Transit_).

Model	Parameters	Estimates	SE	*p*	R^2^	ΔAICc	AICc weight
5	Intercept	0.16	0.69	0.820	0.63	0.00	0.185
	Swim.Intens._Total_	2.5 10^−5^	4.1 10^−6^	< 10^−5^			
	Species (NFS)	1.33	0.54	0.022			
6	Intercept	0.15	0.67	0.824	0.67	0.48	0.146
	Swim.Intens._Total_	5.0 10^−5^	1.7 10^−5^	0.007			
	Stroke.Count_Total_	−1.4 10^−5^	8.9 10^−6^	0.138			
	Species (NFS)	1.97	0.67	0.008			
7	Intercept	−0.20	0.71	0.775	0.66	0.82	0.123
	Stroke.Count_Dive_	4.3 10^−5^	1.5 10^−5^	0.008			
	Swim.Intens._Transit_	1.9 10^−5^	5.7 10^−6^	0.003			
	Species (NFS)	0.96	0.51	0.075			
8	Intercept	0.46	0.65	0.487	0.61	1.52	0.087
	Stroke.Count_Dive_	4.2 10^−5^	1.5 10^−5^	0.011			
	Swim.Intens._Transit_	1.7 10^−5^	5.9 10^−6^	0.008			
9	Intercept	−0.02	0.71	0.920	0.65	1.78	0.076
	Stroke.Count_Transit_	7.7 10^−6^	2.7 10^−6^	0.010			
	Swim.Intens._Dive_	6.0 10^−5^	1.7 10^−5^	0.002			
	Species (NFS)	1.53	0.58	0.016			
10	Intercept	0.95	0.67		0.50	4.63	0.045
	Stroke.Count_Total_	1.2 10^−5^	2.510^−6^				

Only models with ΔAICc < 2 and the model with only Stroke.Count_Total_ as an explanatory variable (simplest model for comparison) are presented.
